# COVID-19 and Sarcoidosis, Readiness for Vaccination: Challenges and Opportunities

**DOI:** 10.3389/fmed.2021.672028

**Published:** 2021-04-30

**Authors:** Michael Manansala, Amit Chopra, Robert P. Baughman, Richard Novak, Elyse E. Lower, Daniel A. Culver, Peter Korsten, Wonder P. Drake, Marc A. Judson, Nadera Sweiss

**Affiliations:** ^1^Department of Medicine, Academic Internal Medicine, University of Illinois at Chicago, Chicago, IL, United States; ^2^Division of Pulmonary and Critical Care Medicine, Department of Medicine, Albany Medical College, Albany, NY, United States; ^3^Department of Medicine, University of Cincinnati Medical Center, Cincinnati, OH, United States; ^4^Division of Infectious Disease, Department of Medicine, University of Illinois at Chicago, Chicago, IL, United States; ^5^Cleveland Clinic, Department of Pulmonary Medicine, Cleveland, OH, United States; ^6^Department of Nephrology and Rheumatology, University Medical Center Göttingen, Göttingen, Germany; ^7^Division of Infectious Disease, Department of Medicine, Vanderbilt University School of Medicine, Nashville, TN, United States; ^8^Division of Rheumatology, Department of Medicine, University of Illinois at Chicago, Chicago, IL, United States

**Keywords:** coronavirus – COVID-19, sarcoidosis, vaccination, immunosuppression, SARS-CoV-2

## Abstract

Sarcoidosis is an immune mediated chronic inflammatory disorder that is best characterized by non-caseating granulomas found in one or more affected organs. The COVID-19 pandemic poses a challenge for clinicians caring for sarcoidosis patients who may be at increased risk of infection compared to the general population. With the recent availability of COVID-19 vaccines, it is expected that clinicians raise questions regarding efficacy and safety in sarcoidosis. However, studies examining safety and efficacy of vaccines in sarcoidosis are lacking. In this review, we examine the current literature regarding vaccination in immunocompromised populations and apply them to sarcoidosis patients. The available literature suggests that vaccines are safe and effective in patients with autoimmune disorders and in those taking immunosuppressive medications. We strongly recommend the administration of COVID-19 vaccines in patients with sarcoidosis. We also present a clinical decision algorithm to provide guidance on vaccination of sarcoidosis patients against COVID-19.

## Introduction

Sarcoidosis is an immune-mediated chronic inflammatory disorder characterized by the formation of non-caseating granulomas in one or more affected organs. These granulomas are hypothesized to be the result of a maladaptive inflammatory response to an unknown antigen in individuals who are genetically susceptible (1, 2). Sarcoidosis most commonly affects the lungs in more than 90% of patients, resulting in potentially diminished baseline pulmonary function. Unsurprisingly, respiratory failure is the leading cause of mortality in these patients (3). However, sarcoidosis can also have cardiac, neurologic, dermatologic, and ophthalmic manifestations, among others (1, 3). The COVID-19 pandemic poses a challenge for all physicians, but especially those caring for patients with sarcoidosis; a large proportion of these patients require immunosuppressive medications, which can lead to an increased risk for severe and opportunistic infections compared to the general population (4).

Since the emergence of the severe acute respiratory syndrome coronavirus 2 (SARS-CoV-2), the vector responsible for COVID-19, over 130 million cases have been reported globally with over 2.8 million deaths as of April 2021 (5). Given the highly infectious nature of the virus, there has been a swift urgency to develop vaccines against COVID-19. There are over 200 vaccine candidates currently under development (5–7). In light of the emergency use authorizations granted by the US Food and Drug Administration (FDA) for several COVID-19 vaccines, we aim to elucidate the unique challenges posed by vaccination against COVID-19 in sarcoidosis patients. At present, no data is available regarding the safety and efficacy of COVID-19 vaccination in this population. In this review we present our own clinical approach to vaccination in sarcoidosis patients with the aim of providing expert-based recommendations to physicians and patients facing sarcoidosis.

## COVID-19 and Sarcoidosis

Our knowledge of the pathophysiology of COVID-19 has evolved at a rapid pace since the start of the pandemic. SARS-CoV-2 targets the ACE2 receptor found in the nasopharynx, oropharynx, and lung through its numerous viral structural spike (S) proteins. After SARS-CoV-2 infiltrates the body, it rapidly replicates, spreads, and in some cases, can cause significant organ damage (8). Although most patients with COVID-19 exhibit asymptomatic or mild disease, a small subset experiences a severe presentation associated with increased mortality. It is thought that much of the morbidity and mortality observed with COVID-19 stems from a hyperactive immune response that is not sufficiently regulated. This phenomenon is known as cytokine storm and has also been observed with other types of coronavirus, including severe acute respiratory syndrome (SARS) and Middle Eastern respiratory syndrome (MERS). Cytokine storm can lead to severe symptoms including acute respiratory distress syndrome (ARDS), sepsis, and multiorgan failure (9).

It is inconclusive based on the current available literature if patients with sarcoidosis are at increased risk of contracting COVID-19. A study by Baughman et al., in which risks and outcomes of COVID-19 in sarcoidosis patients were evaluated via self-reported questionnaire, found that the overall rate of COVID-19 was 2.23% or 22,308 cases per million from April to July 2020. Notably, this was higher than the rate of 1,060 cases per million in the USA at the time. However, a subset analysis comparing rates of COVID-19 in sarcoidosis patients and the general population in the same locality over the same time period revealed the rate of infection was similar in both groups (10). In another cohort of 238 sarcoidosis patients, the rate of COVID-19 was 2.1% from March to April 2020 (11). Without prospective data, it is difficult to draw firm conclusions about the risk of COVID-19 in sarcoidosis as there is a large variation in the rate of infection based on geography as well as the time period of observation.

The current literature suggests that patients with sarcoidosis are at increased risk for worse COVID-19 outcomes. In the same study by Baughman et al., it was found that the rate of hospitalization for sarcoidosis patients with COVID-19 was 15.8%, with one third of those patients requiring ICU care (10). Additionally, there are several factors that can potentially increase the risk of mortality from COVID-19 in sarcoidosis. Firstly, sarcoidosis patients with lung involvement may have decreased pulmonary reserve, which may increase their risk of respiratory failure upon contracting COVID-19 (12). Indeed, Morgenthau et al. found that sarcoidosis patients with moderate to severely decreased lung function experienced greater rates of intubation and in hospital mortality compared to the general population (13). Additionally, comorbidities traditionally associated with glucocorticoid use—such as hypertension, diabetes, and obesity—are more prevalent in patients with sarcoidosis and are independent risk factors for worse COVID-19 outcomes (14, 15). It is apparent there is a need to protect this population from poor COVID-19 outcomes via vaccination.

## Vaccinations in Sarcoidosis

There is limited and conflicting data on the efficacy of vaccination in sarcoidosis patients. In a small case-control study involving 48 sarcoidosis patients and 33 healthy controls, it was found that 50% of sarcoidosis patients did not mount an adequate antibody response to diphtheria vaccination compared to 23% of healthy controls (16). Conversely, Tavana and colleagues performed a prospective study comparing 23 sarcoidosis patients to 26 healthy controls and measured serologic response to influenza vaccination. They found that the serologic response to multiple influenza antigens (H1N1, H3N2, and B) was comparable between both groups (17). However, both studies failed to clarify if patients in the sarcoid cohorts were receiving immunosuppressive medications. Thus, the efficacy of vaccination in sarcoidosis has not been clearly established.

A major concern regarding the efficacy of vaccination in sarcoidosis is the impact of immunosuppressive medications on mounting an appropriate immune response. Of particular interest are glucocorticoids, which are first-line for treatment of sarcoidosis. Other agents include methotrexate, commonly utilized as a steroid-sparing alternative, and biologic medications such as TNF-alpha inhibitors and B-cell depleting therapy, which may be necessary for refractory disease (18). To provide guidance on vaccination practices in sarcoidosis, we extrapolate data from patients with rheumatoid arthritis (RA) as this issue has been studied extensively in this disease.

A meta-analysis of 13 studies involving 886 RA patients evaluated rates of seroprotection, which is defined as attainment of antibody titers above a predetermined level, after influenza vaccination. The rates of seroprotection were similar between RA patients on glucocorticoids and healthy controls (19). Other immunosuppressive agents have been shown to decrease rates of seroprotection. In a cohort of 219 RA patients who received influenza vaccination, patients on methotrexate (MTX) and TNF-alpha inhibitors demonstrated an adequate, although diminished, antibody response (20). In a randomized control trial, Park et al. found that discontinuation of MTX 2 weeks after vaccination resulted in a statistically significant increase in rate of seroprotection after influenza vaccination without a relevant number of increased RA flares (21). Recent studies show that RA patients on the B cell depleting agent rituximab (RTX) demonstrate a decreased antibody response to influenza vaccination (22, 23). However, Van assen et al. found that RA patients who were vaccinated 6 months after receiving RTX had higher antibody titers compared to those who were vaccinated sooner than 6 months after administration of RTX. This suggests that timing of vaccination is an important factor to consider in patients who are receiving immunosuppressive medications (23). Overall, the available data in RA patients suggest that some immunosuppressive medications negatively impact antibody titers after vaccination while others may not. However, the relationship between antibody titers and protection from infection is not straightforward as higher antibody titers do not necessarily indicate a higher level of protection. Other factors, such as the neutralizing activity of the antibodies produced, are important determinants of protection.

The European League Against Rheumatism (EULAR) offers several salient guiding principles regarding the vaccination of patients with autoimmune inflammatory diseases: (1) vaccination should be administered during periods of quiescent disease, (2) vaccines should ideally be administered prior to planned immunosuppression and (3) non-live vaccines can be administered safely while patients are on systemic glucocorticoids and disease-modifying antirheumatic drugs (24). Regarding safety, the current literature thus far suggests vaccination of patients with autoimmune inflammatory disease with non-live vaccines did not result in exacerbation of disease. Additionally, the panel found that patients did not experience significantly greater frequency of adverse events compared to immunocompetent hosts (25). Given the limited data concerning the efficacy of vaccinations in sarcoidosis, Syed and colleagues extrapolated evidence from vaccination of immunosuppressed populations in order to propose general vaccination recommendations for sarcoidosis patients. Essentially, their recommendation is to administer inactivated vaccines—including pneumococcal, influenza, and hepatitis B vaccines—regardless of the patient's current immunosuppressive regimen. Live attenuated vaccines should be administered prior to initiating any biologic therapy and should be avoided if the patient has already initiated biologic therapy. Of note, these recommendations have been endorsed by the World Association of Sarcoidosis and other Granulomatous Disorders (26).

## Challenges of COVID-19 Vaccination in Sarcoidosis

Vaccine development is a process that typically takes anywhere from 10 to 15 years under usual circumstances. However, a COVID-19 vaccine was granted emergency use authorization in the USA by the Food and Drug Administration (FDA) on December 11, 2020, approximately 12 months after the first reported case (27). The remarkable speed of vaccine development was made possible by a number of factors, including knowledge of the spike protein from prior coronavirus outbreaks and its importance in inducing neutralizing antibodies, the ability to perform multiple phases of clinical trials in parallel rather than in sequence, and the advancement of nucleic acid vaccine technologies (6, 28). There are currently over 200 potential COVID vaccines in development (7).

Besides availability, the largest challenges facing COVID-19 vaccination in sarcoidosis are safety and efficacy. The mRNA COVID-19 vaccines, which currently have the most extensive data and are currently being distributed most widely, demonstrated exceptional efficacy in phase III trials (95 and 94.1% for Pfizer and Moderna vaccines, respectively) (29, 30). Phase III trials will be ongoing for up to 2 years to determine duration of seroprotection. It must be noted that patients with sarcoidosis and other autoimmune disorders, as well as those taking immunosuppressive medication, were excluded from clinical trials. Thus, at this time there is no published data on the efficacy of mRNA vaccines in patients with sarcoidosis given the novelty of mRNA vaccination technology. Currently, the Center for Disease Control (CDC) recommends administration of COVID-19 mRNA vaccines to patients with autoimmune disorders or those taking immunosuppressive medications as long as they do not have any other contraindications to vaccination otherwise (31).

Phase III trials show a favorable safety profile for both COVID-19 mRNA vaccines, characterized mainly by short-term, mild-to-moderate pain at the injection site, fatigue, and headache. Serious systemic adverse events were rare for both vaccines and included mostly fevers, fatigue, myalgias, and arthralgias (29, 30). Phase III trials are planned to continue for a total of 2 years to continue monitoring for adverse events. Particularly important in sarcoidosis is concern for disease flare after vaccination. It is reassuring that a prior study of influenza vaccination in a small sample of sarcoidosis patients did not note any disease flare-up immediately after vaccination as well as during a 6 month follow-up period (17). However, close follow-up of sarcoidosis patients will be vital to observe if vaccination against COVID-19 may potentially precipitate a disease flare.

Another safety concern with the novel vaccines is a phenomenon known as vaccine-associated disease enhancement (VAED), in which the immune response to the vaccine may be causally linked to more severe adverse outcomes upon infection compared to infection without prior vaccination. This is a phenomenon which has rarely been encountered with existing vaccines or viral infections, but should be kept in mind with any new vaccine (7). Phase III trial data from both vaccine studies is reassuring in that the frequency of severe cases of COVID-19—defined as confirmed COVID-19 in addition to severe systemic illness including respiratory failure, shock, or other forms of end-organ damage—were rare in the experimental groups (29, 30).

**Figure 1:**
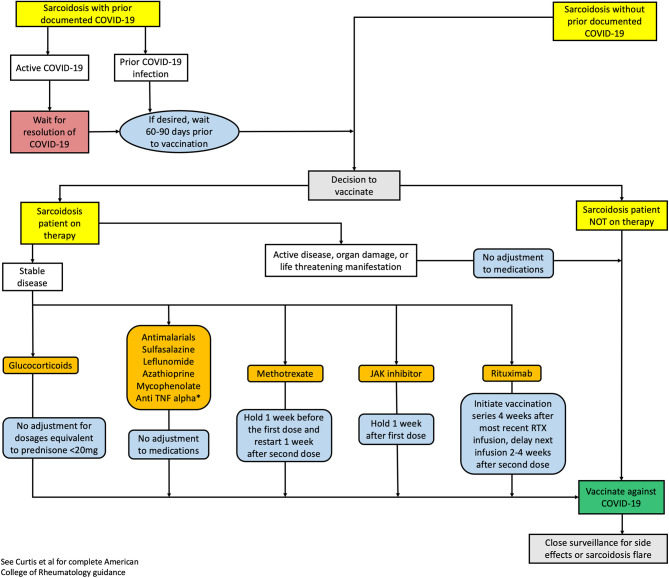
Proposed clinical decision algorithm vaccination in sarcoidosis with or without prior COVID-19 infection. Recommendations are based on ACR guidance for COVID-19 vaccination in patients with rheumatic and musculoskeletal diseases (32) RTX, rituximab; TNF, tumor necrosis factor. *Current guidance recommends no adjustment in patients on anti-TNF alpha therapy. However, recent data suggests that in some patients on infliximab, the antibody response to SARS-CoV-2 may be blunted (33). At this time, it is unknown if this is applicable to sarcoidosis patients.

In the face of these challenges, it is advised to follow the current CDC recommendations and recent guidance from the American College of Rheumatology (ACR) to vaccinate patients with rheumatic diseases in the absence of any contraindications (31, 32). As an overarching principle, vaccinations should be administered prior to planned immunosuppression if clinically possible. Adjusting immunosuppressive therapy to optimize vaccination response should only be done during periods of well-controlled disease. There is limited data regarding the optimal adjustment in immunosuppressive therapy in patients with either active or stable sarcoidosis. Furthermore, the ACR recommends that regardless of disease activity and severity—except in the case of life threatening disease—COVID-19 vaccination should occur as soon as feasible (32). Herein, we propose an algorithm to provide guidance to clinicians regarding COVID-19 vaccination in sarcoidosis patients (Figure 1). At this time, this algorithm is applicable only to two-dose COVID-19 vaccine series. Of particular note, our current algorithm recommends no adjustment to anti-TNF alpha therapy. However, recent data has emerged that in inflammatory bowel disease patients on infliximab, the antibody response to SARS-CoV-2 may be blunted (33). It is currently unknown if this is applicable to sarcoidosis patients and recommendations may evolve as data becomes available. Lastly, although the algorithm is based on recommendations from the ACR, we emphasize that the decision to provide vaccination and temporarily adjust immunosuppressive regimens in sarcoidosis should be individualized for each patient after appropriate counseling and discussion of the risks and benefits. Additional research is required to better understand the safety and efficacy of the various COVID-19 vaccines in sarcoidosis patients with or without immunosuppressive therapy.

## Opportunities

The COVID-19 pandemic offers unique opportunities to develop a greater understanding of novel vaccine technologies in patients with sarcoidosis and other diseases with immune dysfunction; aside from mRNA vaccines, DNA based vaccines are also currently being developed (6). Additionally, we can improve our understanding of the effect of immunosuppressive agents on antibody production and vaccine efficacy.

COVID-19 may also offer valuable insights into immunologic mechanisms implicated in sarcoidosis: autophagy is essential for the clearance of bacterial, viral, and inorganic cellular debris. Prior studies have found the spontaneous formation of sarcoidosis-like granulomas in mouse models with knock-out mutations involving rapamycin activation in macrophages, suggesting a role for autophagy in the pathophysiology of sarcoidosis (34). Recent work by Calendar et al. has identified several mutations in genes involved in the regulation of autophagy in familial forms of sarcoidosis (35). Autophagy may also be important in the pathophysiology of COVID-19. It is posited that coronaviruses take advantage of autophagy and bypass regulatory mechanisms in order to facilitate entry into cells (36). Microscopic examination of lung tissue of COVID-19 patients reveals clusters of multinucleated giant cells and other inflammatory cells reminiscent of those found in sarcoidosis (37). Thus, Calendar et al. hypothesize that the dysfunction in autophagy in sarcoidosis patients decreases trafficking of viral RNA into vesicles during viral infections. It is possible that pathogenic variants in these genes that regulate autophagy may predispose some patients to severe presentations of COVID-19 despite the lack of comorbidities (38). However, further study is needed before this is confirmed.

Lastly, the current pandemic may allow us to improve health disparities regarding vaccination administration and readiness among different populations. Compared with the rate of COVID-19-related deaths among non-Hispanic White individuals (mortality rate, 38/100,000) and adjusting for age, the mortality rate relative to population size is 3.4-fold higher among Black individuals (mortality rate, 131/100,000), 3.3-fold higher among Indigenous and Latino communities (mortality rate for both, 125/100,000), 2.9-fold higher among Pacific Islander individuals (mortality rate, 111/100,000), and 1.3-fold higher among Asian populations (mortality rate, 50/100,000) (39). Women and minorities are more likely to have more severe forms of sarcoidosis, yet distrust in the administration of vaccinations remain. Data confirms that minority populations are taking the COVID-19 vaccination at a significantly lower rate than Caucasians, despite having a higher mortality rate. This follows vaccination patterns seen in influenza (40). We may use this opportunity to evaluate sarcoidosis patients' attitudes regarding readiness for vaccination. For influenza, the rate of vaccination has been found to increase with patient-specific focused education (41). For sarcoidosis patients, more information regarding vaccination rate, patient willingness to receive the vaccines, and the effect of race, gender, and age are needed to provide better patient-centered education. A patient directed online questionnaire is collecting this information. Patients are encouraged to complete the questionnaire at https://redcap.research.cchmc.org/surveys/?s=TJXWAK4FCJ.

## Conclusion

The safety and efficacy of COVID-19 vaccination in sarcoidosis is yet to be determined. However, in light of the severity of the COVID-19 pandemic and the increased risk of severe pulmonary outcomes in sarcoidosis, we strongly recommend that patients with sarcoidosis receive COVID-19 vaccination. Prior studies have shown that vaccines are effective and safe in patients with autoimmune diseases and in those taking immunosuppressive medications. It is important to be cognizant of each patient's immunosuppressants and to counsel patients on risks and benefits of adjusting immunosuppressive regimens in preparation for COVID-19 vaccination. As immunocompromised patients were excluded in the trials for COVID-19 vaccines, careful surveillance must be continued to confirm efficacy and determine the safety profile in these patients. While there are no data available for specific populations with autoimmune disease, the ACR and EULAR have issued practice recommendations for immunocompromised patients (25, 32).

The COVID-19 pandemic offers several opportunities especially in furthering our knowledge of immune responses to vaccination in patients taking immunosuppressives. Additionally, COVID-19 offers a model to allow us to better understand molecular mechanisms that may be implicated in sarcoidosis. We must also use this opportunity to improve attitudes toward vaccination by listening to patient concerns and with each clinical interaction present additional safety and efficacy data to our patients, especially in minority populations.

## Author Contributions

MM wrote and edited the manuscript. MM and NS created the figure. RB, MJ, PK, AC, RN, EL, DC, and WD provided comments and suggestions. All authors contributed to the article and approved the submitted version.

## Conflict of Interest

The authors declare that the research was conducted in the absence of any commercial or financial relationships that could be construed as a potential conflict of interest.

## References

[1] Bargagli E Prasse A. Sarcoidosis: a review for the internist. Int Emerg Med. (2018) 13:325–31. 10.1007/s11739-017-1778-6 29299831

[2] Moller DR Chen ES. Genetic basis of remitting sarcoidosis: triumph of the trimolecular complex? Am J Respir Cell Mol Biol. (2002) 27:391–5. 10.1165/rcmb.2002-0164PS 12356571

[3] Spagnolo P Rossi G Trisolini R Sverzellati N Baughman RP Wells AU. Pulmonary sarcoidosis. Lancet Respir Med. (2018) 6:389–402. 10.1016/S2213-2600(18)30064-X 29625772

[4] Duréault A Chapelon C Biard L Domont F Savey L Bodaghi B. Severe infections in sarcoidosis: incidence, predictors and long-term outcome in a cohort of 585 patients. Medicine. (2017) 96:e8846. 10.1097/MD.0000000000008846 29245251 PMC5728866

[5] WHO. WHO Coronavirus Disease (COVID-19) Dashboard (2020). Available from: https://covid19.who.int/ (accessed April 1, 2021).

[6] Kaur SP Gupta V. COVID-19 vaccine: a comprehensive status report. Virus Res. (2020) 288:198114. 10.1016/j.virusres.2020.198114 32800805 PMC7423510

[7] Haynes BF Corey L Fernandes P Gilbert PB Hotez PJ Rao S. Prospects for a safe COVID-19 vaccine. Sci Transl Med. (2020) 12:eabe0948. 10.1126/scitranslmed.abe0948 33077678

[8] Wiersinga WJ Rhodes A Cheng AC Peacock SJ Prescott HC. Pathophysiology, transmission, diagnosis, and treatment of coronavirus disease 2019 (COVID-19). JAMA. (2020) 324:782. 10.1001/jama.2020.12839 32648899

[9] Ye Q Wang B Mao J. The pathogenesis and treatment of the ‘Cytokine Storm' in COVID-19. J Infect. (2020) 80:607–13. 10.1016/j.jinf.2020.03.037 32283152 PMC7194613

[10] Baughman RP LE Buchanon M Rottoli P Drent M Sellares J Terwiel M. Risk and outcome of COVID-19 infection in sarcoidosis patients: results of a self-reporting questionnaire. Sarcoidosis Vasc Diffuse Lung Dis. (2020) 37:e2020009. 10.36141/svdld.v37i4.10726 33597796 PMC7883514

[11] Manansala M Ascoli C Alburquerque AG Perkins D Mirsaedi M Finn P. Case series: COVID-19 in African American patients with sarcoidosis. Front Med. (2020) 7:588527. 10.3389/fmed.2020.588527 33251236 PMC7672207

[12] Zhou F Yu T Du R Fan G Liu Y Liu Z. Clinical course and risk factors for mortality of adult inpatients with COVID-19 in Wuhan, China: a retrospective cohort study. Lancet. (2020) 395:1054–62. 10.1016/S0140-6736(20)30566-3 32171076 PMC7270627

[13] Morgenthau AS Levin MA Freeman R Reich DL Klang E. Moderate or severe impairment in pulmonary function is associated with mortality in sarcoidosis patients infected with SARS-CoV-2. Lung. (2020) 198:771–5. 10.1007/s00408-020-00392-9 32915271 PMC7484928

[14] Garg S Kim L Whitaker M O'Halloran A Cummings C Holstein R. Hospitalization rates and characteristics of patients hospitalized with laboratory-confirmed coronavirus disease 2019 — COVID-NET, 14 States, March 1–30, 2020. MMWR Morbidity and Mortality Weekly Report. (2020) 69:458–64. 10.15585/mmwr.mm6915e3 32298251 PMC7755063

[15] Sweiss NJ Korsten P Syed HJ Syed A Baughman RP Yee AMF. When the game changes. Chest. (2020) 158:892–5. 10.1016/j.chest.2020.04.033 32360495 PMC7189863

[16] Seyhan EC Günlüoglu G Altin S Cetinkaya E Sökücü S Uzun H. Results of tetanus vaccination in sarcoidosis. Sarcoidosis Vasc Diffuse Lung Dis. (2012) 29:3–10. 23311117

[17] Tavana S Argani H Gholamin S Razavi S-M Keshtkar-Jahromi M Talebian AS. Influenza vaccination in patients with pulmonary sarcoidosis: efficacy and safety. Influenza Other Respir Viruses. (2012) 6:136–41. 10.1111/j.1750-2659.2011.00290.x 21955954 PMC4942082

[18] El Jammal T Jamilloux Y Gerfaud-Valentin M Valeyre D Sève P. Refractory sarcoidosis: a review. Ther Clin Risk Manag. (2020) 16:323–45. 10.2147/TCRM.S192922 32368072 PMC7173950

[19] Huang Y Wang H Tam WWS. Is rheumatoid arthritis associated with reduced immunogenicity of the influenza vaccination? A systematic review and meta-analysis. Curr Med Res Opin. (2017) 33:1901–8. 10.1080/03007995.2017.1329140 28489423

[20] Kapetanovic MC Kristensen L-E Saxne T Aktas T Mörner A Geborek P. Impact of anti-rheumatic treatment on immunogenicity of pandemic H1N1 influenza vaccine in patients with arthritis. Arthrit Res Therapy. (2014) 16:R2. 10.1186/ar4427 24383620 PMC3978632

[21] Park JK Lee YJ Shin K Ha Y-J Lee EY Song YW. Impact of temporary methotrexate discontinuation for 2 weeks on immunogenicity of seasonal influenza vaccination in patients with rheumatoid arthritis: a randomised clinical trial. Ann Rheumat Dis. (2018) 77:898–904. 10.1136/annrheumdis-2018-213222 29572291 PMC5965360

[22] Oren S Mandelboim M Braun-Moscovici Y Paran D Ablin J Litinsky I. Vaccination against influenza in patients with rheumatoid arthritis: the effect of rituximab on the humoral response. Ann Rheumat Dis. (2008) 67:937–41. 10.1136/ard.2007.077461 17981914

[23] Van Assen S Holvast A Benne CA Posthumus MD Van Leeuwen MA Voskuyl AE. Humoral responses after influenza vaccination are severely reduced in patients with rheumatoid arthritis treated with rituximab. Arthrit Rheumat. (2010) 62:75–81. 10.1002/art.25033 20039396

[24] Rondaan C Furer V Heijstek MW Agmon-Levin N Bijl M Breedveld FC. Efficacy, immunogenicity and safety of vaccination in adult patients with autoimmune inflammatory rheumatic diseases: a systematic literature review for the 2019 update of EULAR recommendations. RMD Open. (2019) 5:e001035. 10.1136/rmdopen-2019-001035 31565247 PMC6744079

[25] Furer V Rondaan C Heijstek MW Agmon-Levin N Van Assen S Bijl M. 2019 update of EULAR recommendations for vaccination in adult patients with autoimmune inflammatory rheumatic diseases. Ann Rheumat Dis. (2020) 79:39–52. 10.1136/annrheumdis-2019-215882 31413005

[26] Syed H Ascoli C Linssen CF Vagts C Iden T Syed A. Infection prevention in sarcoidosis: proposal for vaccination and prophylactic therapy. Sarcoidosis Vasc Diffuse Lung Dis. (2020) 37:87–98. 10.36141/svdld.v37i2.9599 33093774 PMC7569559

[27] Ghinai I McPherson TD Hunter JC Kirking HL Christiansen D Joshi K. First known person-to-person transmission of severe acute respiratory syndrome coronavirus 2 (SARS-CoV-2) in the USA. Lancet. (2020) 395:1137–44. 10.1016/S0140-6736(20)30607-3 32178768 PMC7158585

[28] Heaton PM. The covid-19 vaccine-development multiverse. N Engl J Med. (2020) 383:1986–8. 10.1056/NEJMe2025111 32663910 PMC7377255

[29] Polack FP Thomas SJ Kitchin N Absalon J Gurtman A Lockhart S. Safety and efficacy of the BNT162b2 mRNA covid-19 vaccine. N Engl J Med. (2020) 383:2603–15. 10.1056/NEJMoa2034577 33301246 PMC7745181

[30] Baden LR El Sahly HM Essink B Kotloff K Frey S Novak R. Efficacy and safety of the mRNA-1273 SARS-CoV-2 vaccine. N Engl J Med. (2020) 384:403–16. 10.1056/NEJMoa2035389 33378609 PMC7787219

[31] Prevention CfDCa. Interim Clinical Considerations for Use of mRNA COVID-19 Vaccines Currently Authorized in the United States (2020). Available from: https://www.cdc.gov/vaccines/covid-19/info-by-product/clinical-considerations.html#underlying-conditions (accessed February 1, 2021).

[32] Curtis JR Johnson SR Anthony DD Arasaratnam RJ Baden LR Bass AR. American College of Rheumatology Guidance for COVID-19 vaccination in patients with rheumatic and musculoskeletal diseases - version 1. Arthritis Rheumatol. (2021). 10.1002/art.41734. [Epub ahead of print]. 33728796 PMC8250724

[33] Kennedy NA Goodhand JR Bewshea C Nice R Chee D Lin S. Anti-SARS-CoV-2 antibody responses are attenuated in patients with IBD treated with infliximab. Gut. (2021) 70:865–75. 10.1136/gutjnl-2021-324388 33753421

[34] Linke M Pham HTT Katholnig K Schnöller T Miller A Demel F. Chronic signaling via the metabolic checkpoint kinase mTORC1 induces macrophage granuloma formation and marks sarcoidosis progression. Nat Immunol. (2017) 18:293–302. 10.1038/ni.3655 28092373 PMC5321578

[35] Calender A Lim CX Weichhart T Buisson A Besnard V Rollat-Farnier PA. Exome sequencing and pathogenicity-network analysis of five French families implicate mTOR signalling and autophagy in familial sarcoidosis. Eur Respir J. (2019) 54:856–9. 10.1183/13993003.00430-2019 31023854

[36] Fung TS Liu DX. Human coronavirus: host-pathogen interaction. Ann Rev Microbiol. (2019) 73:529–57. 10.1146/annurev-micro-020518-115759 31226023

[37] Tian S Hu W Niu L Liu H Xu H Xiao S-Y. Pulmonary pathology of early-phase 2019 novel coronavirus (COVID-19) pneumonia in two patients with lung cancer. J Thorac Oncol. (2020) 15:700–4. 10.1016/j.jtho.2020.02.010 32114094 PMC7128866

[38] Calender A Israel-Biet D Valeyre D Pacheco Y. Modeling potential autophagy pathways in covid-19 and sarcoidosis. Trends Immunol. (2020) 41:856–9. 10.1016/j.it.2020.08.001 32863134 PMC7416769

[39] Staff ARL. The Color of Coronavirus: COVID-19 Deaths by Race and Ethnicity in the US (2020). Available from: https://www.apmresearchlab.org/covid/deaths-by-race#age (accessed February 15, 2021).

[40] Burger AE Reither EN Mamelund SE Lim S. Black-white disparities in 2009 H1N1 vaccination among adults in the United States: a cautionary tale for the COVID-19 pandemic. Vaccine. (2021) 39:943–51. 10.1016/j.vaccine.2020.12.069 33454136 PMC7800135

[41] Jarrett C Wilson R O'Leary M Eckersberger E Larson HJ. Strategies for addressing vaccine hesitancy - a systematic review. Vaccine. (2015) 33:4180–90. 10.1016/j.vaccine.2015.04.040 25896377

